# New Appliances and Things Medical

**Published:** 1901-02-23

**Authors:** 


### NEW APPLIANCES AND THINGS MEDICAL.

[We shall be glad to receive, at our Office, 28 & 29 Southampton Street, Strand, London, W.C., from the manufacturers, specimens of all new preparations and appliances which may be brought out from time to time.]

### CARNOS INVALID STOUT.

(CARNOS, LIMITED, GREAT GRIMSBY.)

THIS new invalid stout possesses the altogether novel feature of combining the properties of an alcoholic beverage with the nutritive qualities of a meat extract. Carnos, as is well known, contains the soluble constituents of meat prepared on the most approved principles with the presence of albuminoids to the extent of over 20 per cent. The addition of a meat extract of such high merit to a stout of first-rate quality will ensure the invalid who drinks the same the advantages of a tonic, stimulant, and food combined.

### HOSPITAL CHART.

(REYNOLDS AND BRANSON, LIMITED, 13 BRIGGATE, LEEDS.)

Messrs. Reynolds and Branson have sent us a hospital chart which is in use in Dr. Churton's wards in the Leeds General Infirmary. It contains the usual spaces in which various details regarding the daily progress of the case can be conveniently entered. The chief peculiarity of the chart is the arrangement for recording the respiration and pulse rates in the same spaces. The idea is to mark the respirations by a cross and the pulse by a dot in the same chart, and as the numbers for the respiration run from 12 to 42 advancing by threes for each square on the chart, while the numbers for the pulse run from 48 to 168, advancing by twelves to each square (*i.e.*, four times as quickly as the respiration rate), it is suggested that as long as the normal pulse-respiration ratio is maintained the dots and crosses will rise and fall together. Thus with respiration at 18 per minute and the pulse at 72 the dots and crosses will be on the same line; and so they would be at any other level, so long as the assumed normal ratio were maintained; which is all very nice, and any abnormal ratio is "detected at a glance."

There is somewhat of the same dodginess in the arrangement for recording the specific gravity and quantity of the urine. For each of these three lines are provided, all figures within the ordinary range (40 to 60 oz. for quantity, and 1018-1025 for sp. gr.) being placed in the middle spaces, and any above or below the ordinary range being placed on higher or lower levels. Aids to idleness some people might say, but for all that very useful aids to help one in at once "spotting" any divergence from the normal course—or what is assumed to be the normal course.

### NOVELTIES IN SOAP.

(MYNAME SOAP COMPANY, LIMITED, 59 EASTCHEAP, LONDON, E.C.)

THE Myname Soap Company are now introducing a novelty which has very practical applications. Any word or name can be stamped indelibly, so that on each side the lettering remains distinct until it is reduced by use to the thinnest wafer. Apart from the fact that such a method will protect institutions from wholesale robbery, from a medical or surgical point of view it has the advantage that special antiseptic or medicinal soaps can be stamped with the correct descriptive title, so that mistakes will be impossible as long as the soap remains in use. We shall hope to see an extension of this ingenious method for the labelling of all soaps employed in medical or surgical practice.

Another ingenious contrivance introduced by the same firm is the castor-soap powder—which is a fine soap powder contained in a cardboard castor, with a perforated adjustable lid. When intended to be used the lid is turned round so that the perforations are free and the soap powder can be sprinkled on the article to be washed, or on to the water itself.

